# Influenza‐Related Deaths in the Czech Republic Over 21 Seasons

**DOI:** 10.1111/irv.70072

**Published:** 2025-03-06

**Authors:** Jan Kyncl, Marek Brabec, Marek Maly, Vojtech Simka, Ales Urban

**Affiliations:** ^1^ Department of Infectious Diseases Epidemiology National Institute of Public Health Prague Czech Republic; ^2^ Department of Epidemiology and Biostatistics, Third Faculty of Medicine Charles University Prague Czech Republic; ^3^ Department of Biostatistics National Institute of Public Health Prague Czech Republic; ^4^ Institute of Computer Science Czech Academy of Sciences Prague Czech Republic; ^5^ Institute of Atmospheric Physics Czech Academy of Sciences Prague Czech Republic; ^6^ Faculty of Environmental Sciences Czech University of Life Sciences Prague Czech Republic

**Keywords:** excess mortality, influenza, morbidity, mortality

## Abstract

**Background:**

Influenza is a relatively serious infection that causes considerable morbidity and mortality. Epidemics of influenza are reported almost every year.

**Methods:**

Based on the Czech national all‐cause mortality and acute respiratory infection/influenza‐like illness surveillance data for the 1999/2000 to 2019/2020 influenza seasons, excess deaths attributable to influenza were estimated using the threshold derived as 90th percentile of death counts during nonepidemic periods. Daily death counts broken by the 5‐year age intervals were modelled via Poisson generalised additive model.

**Results:**

The estimated total number of excess deaths from influenza during study period was 22,306. Thus, the mean total of excess deaths related to influenza per season was 1062 for the age group 40–94 years. The total number of excess deaths increased steadily with age from the 40–44 age group to the 85–89 age group, which accounted for the highest percentage of excess deaths (17%), followed closely by the 80–84 age group (16%). The age groups 40–44 years and 45–49 years contributed the least (3% each). More than three quarters of excess deaths occurred at age 65 and over (17,027 cases; 76%). Relative numbers of excess deaths per 100,000 population peaked in the oldest age groups of 85–89 and 90–94 years.

**Conclusions:**

We estimate that at least 0.98% of all‐cause mortality throughout the study period was attributable to influenza in the Czech Republic. This excess is not negligible, and public health actions in the field of influenza prevention are vitally needed.

## 1 Introduction

Influenza is an acute viral disease of the respiratory tract that occurs in regular annual epidemic waves of varying magnitude and duration. The disease can present as a mild respiratory infection or as a serious illness requiring hospitalisation, with possible complications and sequelae or death not only in the elderly or chronically ill but also in young healthy persons.

Influenza surveillance in the Czech Republic consists from surveillance of acute respiratory infection (ARI) and surveillance of influenza‐like illness (ILI) and is defined by the 2018 European Union case definition for influenza [1]. The ARI/ILI surveillance is based mainly on epidemiological and clinical surveillance (morbidity reports and mortality statistics of influenza and respiratory infections as well as of all causes) and virological surveillance from the community and hospitals [2, 3].

As influenza infections are typically not laboratory verified, are seldom detected and are typically not noted on hospital discharge forms or death certificates, it is difficult to determine the amount of deaths attributed to seasonal influenza. Another factor that makes it difficult to estimate the seasonal influenza mortality is the fact that symptoms are often subtle and only few people are tested for an active influenza infection. It is also clear that in the death certificates of majority of the deceased in whom influenza likely contributed to death, influenza is not listed as a cause of death [4, 5]. Many deaths linked to influenza can also happen few weeks after resolving the original infection as a result of secondary complications or exacerbations of existing conditions, and in both situations, the influenza viruses are no longer detectable. Therefore, officially reported numbers of influenza deaths in vital statistics usually considerably underestimate the real impact of influenza [4, 5]. To reflect the above, there are several different methods used for estimating the influenza‐associated mortality, and influenza‐related mortality rates have been established in many countries [6, 7, 8, 9, 10, 11, 12]. Overview of statistical and modelling methods used for estimating influenza‐related mortality is presented in [4, 13]. Nevertheless, studies focusing on the Central European population have been rare to date. Although there is a network for European monitoring of excess mortality for public health actions (EuroMOMO), where the statistical model FluMOMO has been developed to estimate influenza‐associated mortality [14], the Czech Republic could not participate in this network because it did not have timely data available.

Our study aims to assess the association between mortality and periods of influenza virus circulation and to determine the excess mortality attributable to influenza. The analysis focuses on the data for the 1999/2000 to 2019/2020 seasons, building on the period covered in the previous study [15]. In addition, the intention is to provide a more detailed insight into the situation in selected adult age groups. In the period currently analysed, the mean population of the Czech Republic was 10.4 million, and the mean annual total number of deaths was 108,300.

## 2 Methods

The study used data for the Czech Republic gathered from the national ARI/ILI surveillance system operated by the Czech National Institute of Public Health, daily all‐cause mortality data provided by the Institute of Health Information and Statistics of the Czech Republic and population data managed by the Czech Statistical Office to calculate excess influenza deaths. Each season began with the 40th calendar week.

To assess the impact of influenza on mortality, it is essential to compare the number of excess deaths with the typical course of the total number of all‐cause deaths. This allows an understanding of the proportion of deaths attributable to influenza. Excess deaths were defined as deaths that exceed the threshold derived from all‐cause deaths during nonepidemic weeks.

High burden of influenza is usually observed in the population of persons over 65 years of age, or in persons over 50 years of age [16]. However, there is an evidence showing that even at the age of 40–49 years, respiratory infections, including influenza, can act as a trigger for serious cardiovascular diseases [17]. Thus, we selected the age group of persons from 40 to 94 years for the assessment. Calculations were made for 5‐year age groups within the range (40–44, 45–49, 50–54, 55–59, 60–64, 65–69, 70–74, 75–79, 80–84, 85–89 and 90–94). This approach allows for an analysis of influenza‐related mortalities and deriving excess influenza deaths for each specific age group separately (based on the same form of the statistical model stratified on age groups). The total number of excess deaths was then calculated as the sum of specific age group contributions. Positive excesses for the period of the 40th–20th calendar week are summed (while negative ones are treated as zeros). This specification has been chosen on the basis of the usual period of influenza circulation in the Northern Hemisphere, in particular in Europe. As the underlying statistical model decomposes the long‐term all‐cause‐mortality (in daily resolution) into long‐term trend and seasonality (yearly periodic) term, as a byproduct, the approach easily identifies seasons with highest and lowest levels of excess deaths, as well as the age groups with the highest and lowest average excess deaths.

### 2.1 Statistical Modelling

Mortality data (death counts by day) broken by the age intervals mentioned above were modelled via Poisson generalised additive model (GAM) [18, 19] with (log) total population of a given age class taken as offset, allowing for flexible (multiple) decomposition into long‐term trend and annual‐periodic seasonal term to model baseline mortality. Both trend and seasonal terms were modelled nonparametrically as complexity‐penalised splines (as the shape of both is a priori unknown, they cannot be modelled via specific parametric terms). The model was fitted simultaneously via maximisation of penalised likelihood [20]. Its general form is $Y_{t}\sim N_{t}.\mathrm{Poi}\left(\mu_{t}\right)$ with

$$\log\left(\mu_{t}\right)=\beta_{0}+s_{T}\left({\text{year}}_{t}\right)+s_{\text{Seas}}\left({\text{yday}}_{t}\right)$$

where

- $Y_{t}$ is the number of deaths in a given age category in day *t*.
- $N_{t}$ is the total population in the age category in day *t*.
- ${\text{year}}_{t}$ is the year of the day *t*.
- ${\text{yday}}_{t}$ is the day within a year (1 for Jan 1; 2 for Jan 2 etc.) of the day *t*.
- $s_{T}$ is a smooth trend function (implemented as cubic spline) to be estimated from data.
- $s_{\text{Seas}}$ is a smooth seasonal component (implemented as cyclic cubic spline to reflect inherent periodicity) to be estimated from data.

**FIGURE 1:**
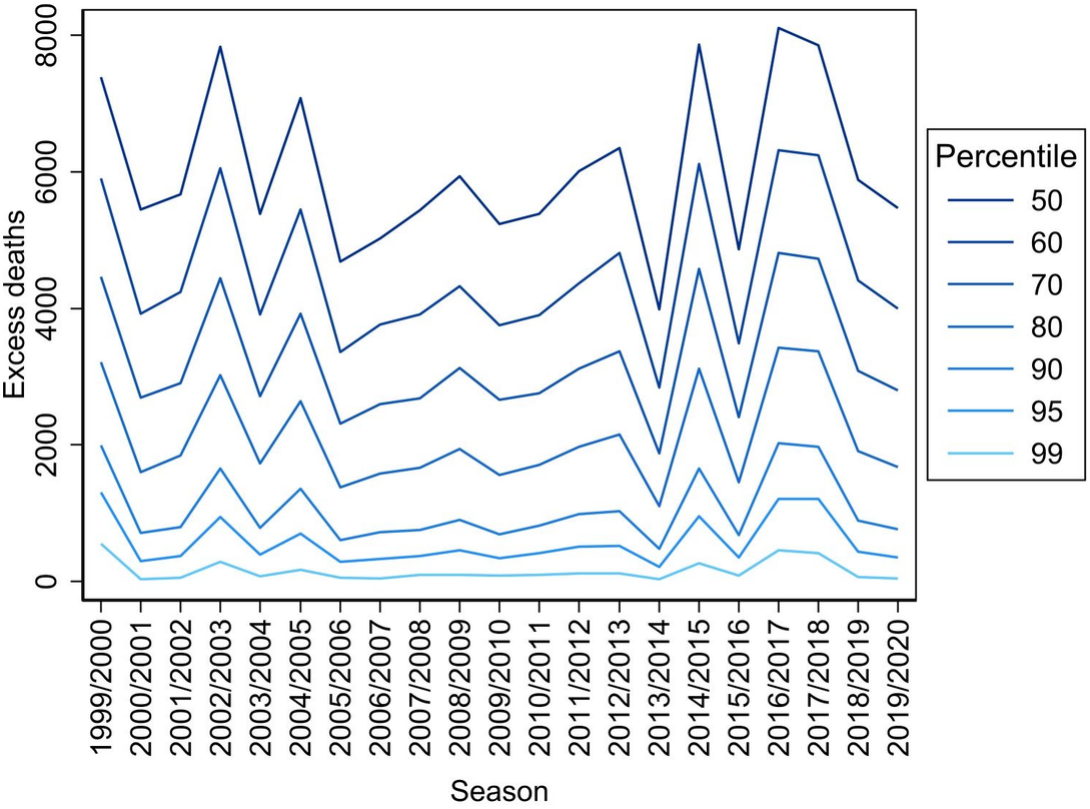
Percentiles of excess deaths exceeding the threshold of all‐cause deaths during nonepidemic periods.

Fitted model gives a (long‐term) ‘baseline mortality’ estimate for a given age category. To flag an elevated mortality (to be attributed to influenza) at day *t*, a threshold needs to be considered. To be empirical (in close touch with the data), we choose the threshold for deaths attributable to influenza as a specific percentile of deaths counts. As shown in Figure 1, we considered selected higher percentiles (specifically 50th, 60th, 70th, 80th, 90th, 95th and 99th percentiles) as potential thresholds as there are no universally accepted rules for selecting a particular percentile that delineates an ‘unusual’ situation from a ‘usual’ one, and it is not clear a priori what percentile should be taken as the threshold. The choice is somewhat ill‐conditioned and should be relegated to the relationship with some relevant external data. Clearly, different percentile choice leads to very different assessments of excess deaths and hence of influenza‐related mortality. To reach empirically some supported guideline for the percentile choice, we considered the Spearman's correlation of each percentile trajectory with the reported total weekly ILI morbidity. For the percentiles specified above, the correlations were 0.38, 0.36, 0.43, 0.43, 0.43, 0.40 and 0.41, respectively. Based on correlation analysis and using published analyses of earlier data periods [15, 21], 90th percentile of excess deaths was chosen for further calculations and outputs. All statistical computations were done within the R environment (R Core Team: R: A Language and Environment for Statistical Computing. Vienna, R Foundation for Statistical Computing, 2024).

## 3 Results

Basic descriptive characteristics of influenza seasons 1999/2000 to 2019/2020 in the Czech Republic are shown in Table 1. Influenza epidemics have occurred in all seasons except one (2013/2014). The duration of each epidemic ranged from 5 to 11 weeks (average 7.25 weeks), with influenza A viruses predominating. The intensity of individual influenza epidemics varied, as evidenced by the maximum ARI and ILI rates.

**TABLE 1:** Characteristics of influenza seasons from 1999/2000 to 2019/2020 in the Czech Republic, rates per 100,000 population.

Season	Epidemic weeks	Duration of epidemic (weeks)	Predominant influenza virus type/subtype	Peak weekly ARI morbidity (per 100,000 population)	Peak weekly ILI morbidity (per 100,000 population)^a^
1999/2000	2–6	5	H3	2803	—
2000/2001	3–8	6	H1	3101	—
2001/2002	47–52	6	H3 + B	1931	—
2002/2003	6–12	7	H3 + B	3313	—
2003/2004	50–7	10	H3	1838	256
2004/2005	6–11	6	H3 + H1	3039	834
2005/2006	7–14	8	B	1613	123
2006/2007	3–8	6	H3	2377	292
2007/2008	50–4	7	H1	1669	178
2008/2009	4–8	5	H3	1837	317
2009/2010	46–51	6	H1	1912	395
2010/2011	3–10	8	H1 + B	1906	369
2011/2012	9–13	5	H3	1329	109
2012/2013	2–10	9	B + H1	1818	352
2013/2014	Nonepidemic	—	H1	1150	40
2014/2015	4–9	6	H3 + B	2020	333
2015/2016	3–13	11	H1 + B	1470	151
2016/2017	51–8	10	H3 + B	1887	323
2017/2018	3–13	11	B	1984	359
2018/2019	4–9	6	H1	1757	230
2019/2020	4–10	7	H3 + B	1896	283

^a^ The ILI reporting started from the 2003/2004 season in relation to the accession of the Czech Republic to the EU.

**FIGURE 2:**
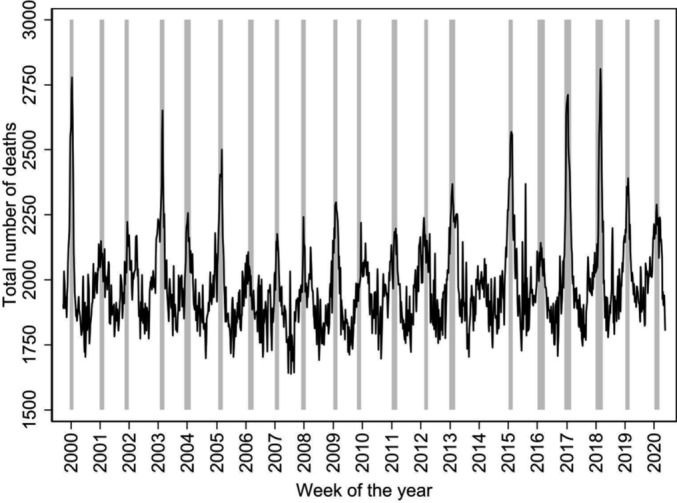
Weekly counts of all‐cause deaths and influenza epidemics weeks, Czech Republic, influenza seasons 1999/2000–2019/2020. Marks on horizontal axis indicate the beginning of the year. Grey highlights indicate influenza epidemic periods.

The course of the weekly counts of all‐cause deaths, with the weeks of the influenza epidemics indicated in grey, is shown in Figure 2.

Based on calculated 90th percentile of deaths above the baseline mortality, the estimated total number of excess deaths attributable to influenza during the 21‐season study period was 22,306. Thus, the mean total of excess deaths related to influenza per season was 1062 for the age group 40–94 years. If we applied this number to the entire population (assuming zero excess deaths for the remaining age groups), we would get an excess rate of 10.2 per 100,000 population. In the context of vital statistics, it means that at least 0.98% of all‐cause mortality throughout the study period was attributable to influenza.

Since very different influenza seasons were observed during the study period, estimates of excess deaths also vary considerably between seasons (Table 2). The highest levels of excess deaths were estimated for the 2016/2017 season (2023 cases; 19.1/100,000 entire population), 1999/2000 season (1995 cases; 19.4/100,000) and 2017/2018 season (1975 cases; 18.6/100,000). Conversely, the lowest excess deaths were observed during 2013/2014 season (481 cases; 4.6/100,000) and 2005/2006 season (605 cases; 5.9/100,000).

**TABLE 2:** Estimated absolute number of excess deaths for each influenza season from 1999/2000 to 2019/2020 in the Czech Republic by age group.

Season	Age group											Total
	40–44	45–49	50–54	55–59	60–64	65–69	70–74	75–79	80–84	85–89	90–94	
1999/2000	33	25	56	79	53	146	217	410	271	486	219	1995
2000/2001	36	55	41	73	40	55	100	111	50	95	58	714
2001/2002	19	25	59	64	33	117	86	114	40	179	62	798
2002/2003	26	53	61	81	101	122	192	304	237	295	186	1658
2003/2004	24	33	43	89	66	82	109	110	85	103	43	787
2004/2005	30	59	53	84	48	96	150	232	308	113	186	1359
2005/2006	28	52	61	46	33	100	66	66	79	22	52	605
2006/2007	21	17	23	74	60	45	86	109	148	47	97	727
2007/2008	31	20	44	67	86	43	87	49	149	89	85	750
2008/2009	21	28	55	53	55	45	62	127	196	169	94	905
2009/2010	34	42	77	54	60	72	59	79	87	98	29	691
2010/2011	40	37	56	81	108	64	82	109	64	109	63	813
2011/2012	22	37	55	106	70	115	86	124	162	148	62	987
2012/2013	22	38	47	67	105	122	65	130	197	181	61	1035
2013/2014	9	28	29	38	46	65	54	31	60	65	56	481
2014/2015	22	20	41	58	110	143	134	163	359	367	241	1658
2015/2016	22	33	48	68	60	70	58	88	96	87	53	683
2016/2017	23	40	44	63	107	151	157	190	406	450	392	2023
2017/2018	43	28	40	68	137	213	262	257	323	399	205	1975
2018/2019	34	37	43	51	117	85	63	132	119	127	86	894
2019/2020	32	23	24	52	66	62	77	120	113	139	60	768
Total	572	730	1000	1416	1561	2013	2252	3055	3549	3768	2390	22,306

The total number of excess deaths increased steadily with age from the 40–44 age group to the 85–89 age group. The 85–89 age group accounted for the highest percentage of excess deaths over the study period, with a total of 3768 (17%), followed closely by the 80–84 age group with 3549 deaths (16%) and, by some distance, the 75–79 and 90–94 age groups (14% and 11%, respectively). The age groups 40–44 years (572 cases; 3%) and 45–49 years (730 cases; 3%) contributed the least to the total number of excess deaths. More than three quarters of excess deaths occurred at age 65 and over (17,027 cases; 76%). The average number of excess deaths per season was 251 in the 40–64 age group and 811 in the 65–94 age group.

If we look at excess deaths through relative numbers per 100,000 population, both in individual seasons and in mean values per season, we see a relatively slow increase with age in the 40–64 age group, which then accelerates, and is steep from the age of 80 (Table 3). Estimated excess mortality peaks in the oldest age group of 90–94 years. If we merge the two categories with the highest rates, we get an excess rate of 204.5 per 100,000 population for the 85–94 age group. In a broader summarisation, the mean relative excess mortality is 48.7 per 100,000 population in the 65–94 age group.

**TABLE 3:** Estimated relative number of excess deaths per 100,000 population for each influenza season from 1999/2000 to 2019/2020 in the Czech Republic by age group.

Season	Age group										
	40–44	45–49	50–54	55–59	60–64	65–69	70–74	75–79	80–84	85–89	90–94
1999–2000	4.6	3.1	7.1	12.9	11.6	32.2	53.3	128.9	228.2	542.5	832.8
2000–2001	5.3	6.9	5.1	11.4	8.5	12.5	24.6	34.3	37.8	112.9	215.2
2001–2002	2.9	3.2	7.3	9.4	6.7	27.5	21.2	35.2	26.0	238.5	225.8
2002–2003	4.1	7.0	7.7	11.3	19.6	29.4	47.6	94.4	132.6	450.5	649.1
2003–2004	3.7	4.5	5.5	12.0	12.1	19.9	27.4	34.3	42.6	174.6	145.2
2004–2005	4.5	8.4	6.8	11.0	8.3	23.0	38.4	72.4	144.6	187.8	626.7
2005–2006	4.1	7.7	7.9	5.9	5.5	23.2	17.3	20.5	36.0	31.8	186.0
2006–2007	3.0	2.6	3.0	9.5	9.4	10.0	23.2	33.5	66.7	57.1	388.1
2007–2008	4.4	3.1	5.9	8.7	12.7	9.0	23.8	15.0	66.6	92.3	384.7
2008–2009	3.0	4.3	7.5	6.9	7.8	8.9	17.0	39.1	86.9	155.5	455.2
2009–2010	4.8	6.2	11.0	7.1	8.3	13.5	15.9	24.7	38.2	84.0	131.2
2010–2011	5.7	5.3	8.3	10.7	14.6	11.4	21.3	34.8	27.7	89.9	241.0
2011–2012	3.0	5.3	8.4	14.2	9.4	19.3	21.3	40.3	69.0	119.3	197.4
2012–2013	2.9	5.4	7.3	9.2	14.3	19.3	15.3	42.8	83.1	143.4	165.5
2013–2014	1.1	4.0	4.5	5.4	6.3	9.9	11.9	10.2	25.3	50.6	134.6
2014–2015	2.6	2.9	6.2	8.5	15.1	21.2	28.0	52.4	152.4	279.3	535.9
2015–2016	2.5	4.7	7.0	10.4	8.3	10.2	11.6	27.2	41.3	64.5	112.4
2016–2017	2.5	5.6	6.3	9.9	15.1	21.9	29.5	55.8	177.1	324.8	804.3
2017–2018	4.6	3.8	5.7	10.9	19.7	31.1	46.3	71.4	141.7	282.7	410.1
2018–2019	3.6	4.7	6.2	8.1	17.3	12.5	10.7	34.4	51.8	89.3	167.5
2019–2020	3.5	2.7	3.5	8.0	10.1	9.2	12.7	29.7	48.0	98.2	113.7
Mean	3.6	4.8	6.6	9.6	11.5	17.5	24.5	44.3	79.8	168.5	333.1

**FIGURE 3:**
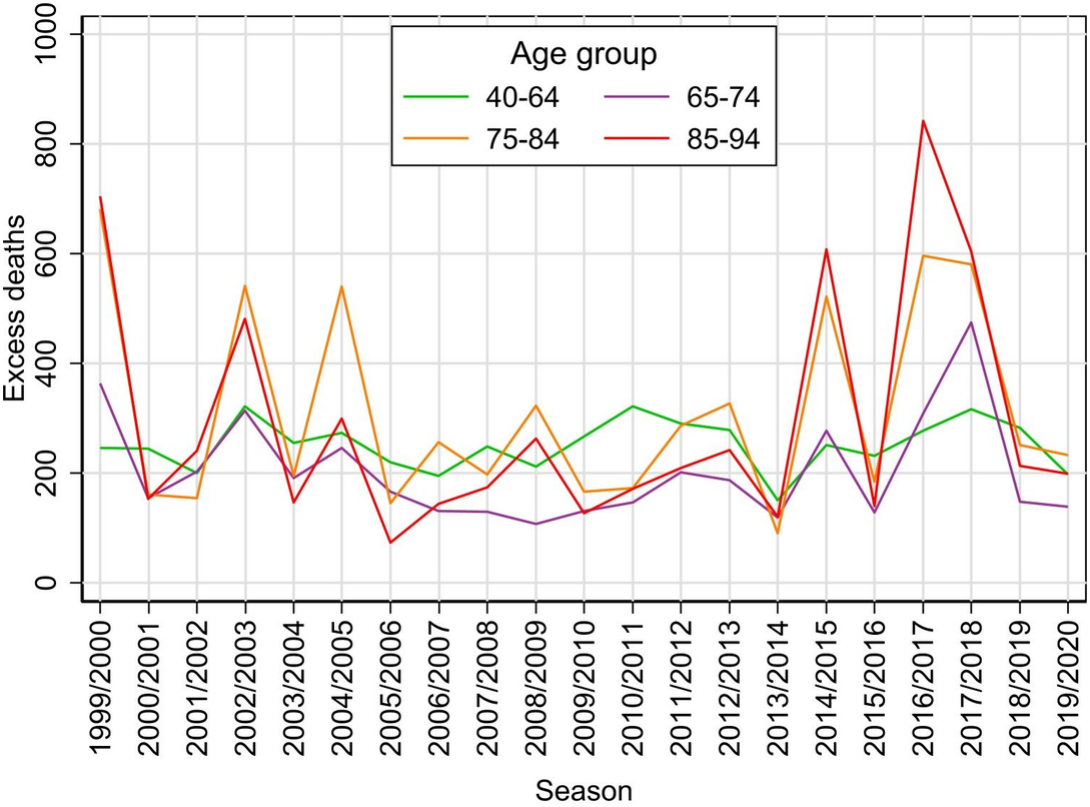
Estimated amount of excess deaths for each influenza season from 1999/2000 to 2019/2020 seasons in the Czech Republic, by aggregated age groups.

**FIGURE 4:**
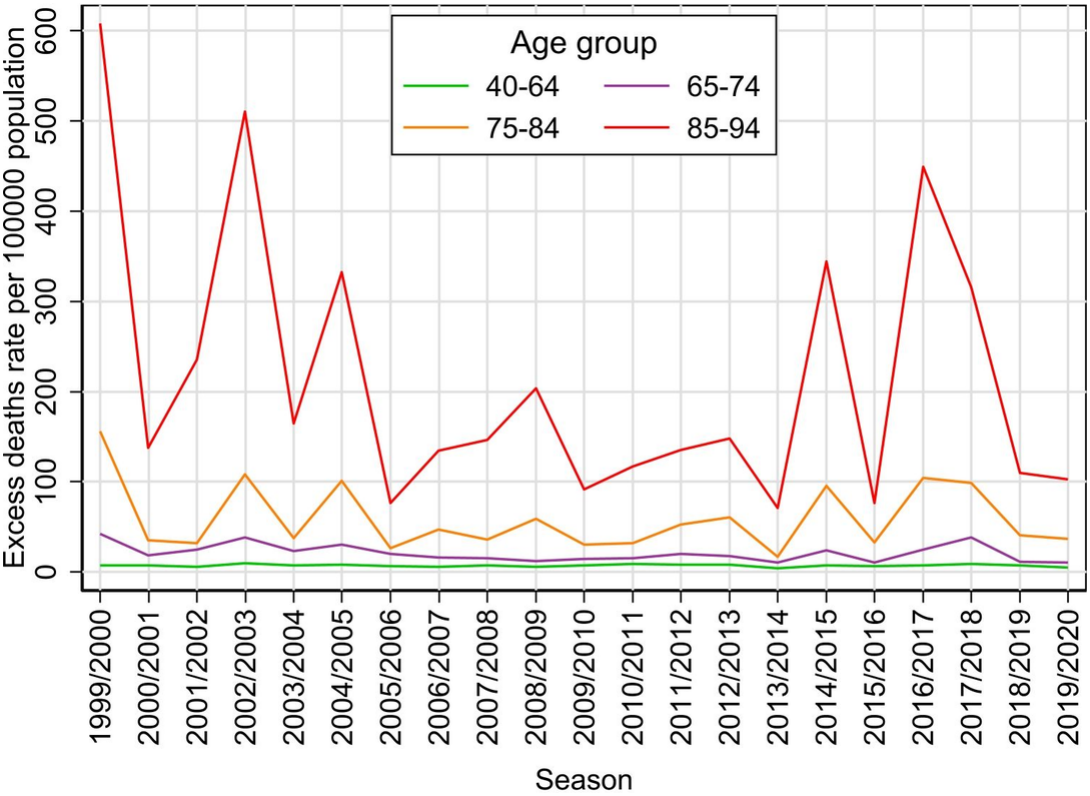
Estimated excess death rates per 100,000 population for each influenza season from 1999/2000 to 2019/2020 seasons in the Czech Republic, by aggregated age groups.

To more clearly present the evolution of the estimates over the seasons, the 5‐year age groups were combined into broader groups of 40–64, 65–74, 75–84 and 85–94. While the absolute numbers of excess deaths are very roughly similar across these groups (Figure 3), the relative numbers show a clear ordering towards higher numbers in the older age groups (Figure 4). Both the absolute and relative counts show that in the seasons more heavily affected by influenza, increases in the number of deaths occur predominantly in the two oldest age groups.

## 4 Discussion

Influenza virus activity is detected in Europe each winter, yet the precise timing and size of this activity is highly unpredictable. The knowledge of the impact of influenza burden is emphasised by the fact that influenza surveillance is one of the oldest infectious disease surveillance programmes coordinated by the World Health Organization [22]. Over time, since Serfling's pioneering work [23], many different methods have been developed to estimate the number of influenza attributable deaths based on total observed deaths [13].

There are also a number of papers that estimate influenza burden by the determination of respiratory deaths or deaths from pneumonia and influenza. This approach is primarily (but not exclusively) used in the United States. Some papers that use the above‐mentioned technique are multicentre studies and, because of the use of a uniform methodology, produce strong results and are highly cited [7, 8]. However, in Europe, a quite common approach is to monitor all‐cause mortality [13, 15, 24, 25, 26, 27, 28, 29]. There are several reasons for such approach, mainly different and not fully harmonised national practices in the reporting of deaths. All‐cause data are easier to obtain than cause‐specific data and not subject to coding bias. The proportion of officially reported deaths from respiratory diseases among all deaths varies considerably between countries of similar economical and health system characteristics (even neighbouring countries) and is low in the Czech Republic, probably for administrative reasons and historical practices. This is probably why the estimates of the excess influenza mortality for the Czech Republic based only on reported respiratory deaths [7, 8] are roughly two to four times lower than estimates based on all‐cause deaths. Similar differences were seen for other countries, as well [30]. For this reason, we based our estimates on the total number of deaths.

Our analysis provides insights into the impact of influenza on mortality over two decades. We found that the mean total of excess deaths related to influenza per season during the study period was 1062 for the age group 40–94 years. It means that approximately 1% of all‐cause mortality throughout the study period was attributable to influenza. In our estimates of the excess, we focused only on age groups that are usually heavily affected by influenza. Also, in the remaining groups, the estimates of baseline mortality would be subject to greater imprecision. Thus, the aforementioned proportion should be interpreted as a lower estimate of the true burden, which might be a potential limitation of the study. However, the difference would be marginal. For instance, a population‐based time‐series study assessing excess mortality from respiratory and cardiovascular diseases found that the group aged ≥ 60 years accounted for 96.6% of total excess mortality [16]. To put our calculated mortality rate attributable to influenza into perspective, data from Demographic Yearbooks of the Czech Republic show very similar death burden (with rate of 1.02%) for all infectious diseases as defined by the Chapter I (Certain infectious and parasitic diseases, codes A00‐B99) of the International Classification of Diseases, 10th revision.

As shown at Figure 2, individual influenza seasons are highly unpredictable. This is a well‐known phenomenon, and it has been noted also in our previous study assessing the time period between 1982 and 2000 [15]. Accordingly, we found substantial differences in excess estimates among individual influenza seasons (with individual seasonal death counts ranging from 481 to 2023), similarly as in other countries [6, 24, 26, 27, 28, 31].

An analysis of influenza mortality in the Czech Republic for the period 1982–2000 estimated that 2.17% of all deaths were attributable to influenza [15]. Such a high proportion may most likely be explained by two severe influenza epidemics during the analysed period. Applying the same statistical procedure to data for 1999–2013, which does not include such large epidemics, resulted in an estimate of 1.31% of all‐cause mortality attributable to influenza [21]. The results from our current analysis over the follow‐up period are consistent with the downward trend observed in mentioned studies. Similar tendency of declining influenza mortality over the year was found also in Denmark [27].

Our estimates of influenza mortality are close to those for Spain, where similar age groups were analysed [28]. In accordance with previous observations, we observed that higher mortality rates occurred during influenza seasons characterised by A/H3N2 predominant influenza virus subtype [15, 32]. However, contrary to what is commonly assumed, the causal influenza virus subtype does not seem to be a major determinant of clinical presentation and severity of influenza illness [33]. Probably the only exception is the different impact of the 2009 A/H1N1 pandemic virus with low total number of influenza deaths and altered involvement of age groups [34]. Similarly, based on data from the Czech national virological surveillance, during the 2009 influenza pandemic and the following season, there were lower numbers of samples positive for influenza in seniors (both numbers and proportions) [3]. Also, higher estimated numbers of excess deaths in the younger age groups (45–54 years) during the 2009/2010 season were observed in our study.

To specify a threshold for estimating the excess of influenza deaths, we chose an approach based on the calculation of percentiles, which allows us to relate to population variability in a meaningful way and thus see explicitly what we are doing. Common approaches often estimate the mean value, which can result in different percentiles in different circumstances. The plot of various percentiles shows that the trend and occurrence of major influenza epidemics are well estimated by a multiplicative model with smooth components for seasonality and trend, and relatively insensitive to the concrete choice of percentile as a threshold (as long as we stay within a reasonable range of, say 60th–95th percentiles). However, the actual quantitative estimate of influenza‐related mortality is obviously highly sensitive to the concrete threshold choice. Although Nunes et al. [31] state that all‐cause excess mortality is a robust indicator of influenza burden, our efforts show that the choice of threshold is ill‐conditioned problem that has so far been largely neglected. Based on anchoring to additional sources of information (primarily ILI data in our case), the 90th percentile of excess deaths was eventually chosen for the analyses. It also appears appropriate because extreme quantiles are less accurate from a statistical perspective.

Excess mortality might be caused not only by influenza but also by other respiratory viruses such as for example RSV [34]. Moreover, also noninfectious factors may play its role, especially low ambient temperature in winter. Our previous research has confirmed that, after the removal of effect of influenza, the influence of the cold spells on mortality exists [35], but even in periods of severe cold spells, the observed number of related excess deaths was rather low.

Besides changes in the age structure of the population, the level of influenza vaccination also may affect mortality. It is known that after the introduction of the nationwide vaccination programme, which led to a significant increase in vaccination coverage in a short period of time, there was a decrease in influenza mortality among Dutch elderly people [36]. In our study, we did not consider the effect of seasonal influenza vaccination as the vaccine coverage was consistently low in the Czech Republic during the study period (accounting for 5%–8% of the total population and around 20%–25% in the elderly population) and varied from year to year approximately by 1%–2% only [37]. Such small changes can be considered insignificant in terms of potential impact on the results.

## 5 Conclusions

The presented results clearly, consistently and unambiguously provide the evidence of nonnegligible excess in death rates related to influenza. We estimate that approximately 1% of all‐cause mortality throughout the study period was attributable to influenza. The 65+ and 50–64 age groups accounted for 76% and 18%, respectively, of the total excess deaths in the Czech Republic. Public health actions in the field of influenza prevention are vitally needed, especially vaccination against influenza in the elderly should be promoted as much as possible.

## Author Contributions

**Jan Kyncl:** conceptualization, writing – original draft, writing – review and editing, supervision, data curation. **Marek Brabec:** writing – review and editing, methodology, formal analysis. **Marek Maly:** writing – original draft, writing – review and editing, supervision, data curation, visualization. **Vojtech Simka:** writing – review and editing, data curation, investigation, validation. **Ales Urban:** writing – review and editing, funding acquisition, validation.

## Conflicts of Interest

The authors declare no conflicts of interest.

### Peer Review

The peer review history for this article is available at https://www.webofscience.com/api/gateway/wos/peer‐review/10.1111/irv.70072.

## Data Availability

The data that support the findings of this study are available from the corresponding author upon reasonable request.
